# Impact of combined ischemic preconditioning and melatonin on renal ischemia-reperfusion injury in rats

**DOI:** 10.22038/IJBMS.2022.67127.14722

**Published:** 2023-02

**Authors:** Hesham AD Abdel-Razek, Mohamed Soliman Rizk, Ghada S Amer, Mona A Kora, Aya M Afifi, Sally S Donia

**Affiliations:** 1 Department of Medical Physiology, Faculty of Medicine, Menoufia University, Shebein El-Koum, Egypt; 2 Department of Medical Biochemistry & Molecular Biology, Faculty of Medicine, Menoufia University, Shebein El-Koum, Egypt; 3 Department of Pathology, Faculty of Medicine, Menoufia University, Shebein El-Koum, Egypt

**Keywords:** Ischemic preconditioning, Ischemia-reperfusion injury, Melatonin, Oxidative stress, Wistar rat

## Abstract

**Objective(s):**

Studying the effect of melatonin pretreatment and ischemic preconditioning on renal ischemia-reperfusion injury (IRI).

**Materials and Methods:**

Forty-eight Wistar rats were randomized into six groups: control, sham operation, IRI (IRI in left kidney + right nephrectomy), IRI+ischemic preconditioning, IRI+Melatonin, and IRI+ischemic preconditioning+Melatonin groups. Melatonin (10 mg/kg) was intraperitoneally injected for 4 weeks before renal IRI. Ischemic preconditioning was performed by three cycles of 2 min-ischemia followed by 5 min-reperfusion period. A right nephrectomy was initially done and the left renal artery was clamped for 45 min. After 24 hr of ischemia-reperfusion, rats were decapitated. Kidney tissue samples were taken for histopathological assessment and the determination of kidney proinflammatory and anti-inflammatory cytokines, apoptotic protein caspase-3, oxidative stress markers, and activities of antioxidant enzymes. Serum creatinine and blood urea nitrogen (BUN) concentrations were measured for evaluation of renal function.

**Results:**

Renal IRI animals showed increased levels of creatinine, BUN, tumor necrosis factor-α (TNF-α), caspase-3, total nitrite/nitrate, and malondialdehyde (MDA), and decreased levels of interleukin-13 (IL-13), and activities of glutathione peroxidase (GPx) and superoxide dismutase (SOD). Melatonin pretreatment or ischemic preconditioning resulted in decreased creatinine, BUN, TNF-α, caspase-3, nitrite/nitrate, and MDA, and increased IL-13, GPx, and SOD, with improved histopathological changes. Combined melatonin and ischemic preconditioning showed more effective improvement in renal IRI changes rather than melatonin or ischemic preconditioning alone.

**Conclusion:**

Combined melatonin and ischemic preconditioning have better beneficial effects on renal IRI than applying each one alone.

## Introduction

Renal ischemia-reperfusion injury (IRI) is a common cause of acute renal failure, renal graft rejection, and renal cell death (1). It is commonly encountered in clinical situations, such as trauma, aortic bypass surgery, hemorrhagic shock, and renal transplantation (2). In fact, renal IRI includes complex inflammatory processes that involve the release of reactive oxygen species (ROS) and proinflammatory cytokines, such as TNF-α. ROS oxidize the amino acids in the nephron, resulting in the loss of important functional properties (3-5), while lipid peroxidation of cell membrane decreases membrane viability, and cleavage and crosslinking of renal DNA occurs leading to harmful mutations (6).

In a trial of tissue adaptation to IRI, the body takes advantage of intrinsic defense mechanisms by ischemic preconditioning (Ipc) that was first described in the heart (7). Although the protective effect of preconditioning against IRI has also been thoroughly described in the liver (8) and kidney (9), the mechanisms of its renoprotective action are poorly understood and not well defined. 

Melatonin is an endogenous hormone mainly synthesized and secreted by the pineal gland which functions as a regulator of sleep, circadian rhythm, and immune function (10). Melatonin and its derivatives have potent antioxidant and anti-inflammatory properties by inhibiting inflammatory cytokines and decreasing prostaglandin E2 (11).

The present study aimed to investigate the possible beneficial effects of melatonin pretreatment for four weeks and ischemic preconditioning on a rat model of renal IRI, as well as the underlying mechanisms behind their effects.

## Materials and Methods

The present study was carried out at the Medical Physiology and Medical Biochemistry & Molecular Biology departments, Faculty of Medicine, Menoufia University, Egypt. The experimental procedures, animal handling, sampling, and sacrification were performed according to The International Ethical guidelines for Investigations of Laboratory Animals and the Guide for The Care and Use of Laboratory Animals (12) and were approved by The Ethical Committee of The Faculty of Medicine, Menoufia University.

***Animals and experimental groups***

Forty-eight male Wistar albino rats (aged 2–3 months, weighing 160–200 g, each) were used. Rats were caged, four per cage, in fully ventilated cages (80 x40 x30 cm), at normal room temperature, under normal light/dark cycle, with free access to water and standard laboratory rat chow. After two weeks of acclimatization, rats were divided randomly into six equal groups (n=8 each): 1) Control (C) group: normal rats in this group were fed with rat chow for 4 weeks. All rats in the other five groups had a right nephrectomy, with a dorsal flank incision after 4 weeks. 2) Sham (S) group: left renal pedicle was surgically exposed for 45 min without induction of ischemia. 3) Renal ischemia-reperfusion injury (IRI) group: induction of definitive ischemia by clamping of the left renal pedicle for 45 min. 4) Renal ischemia-reperfusion injury with ischemic preconditioning (IRI+Ipc) group: ischemic preconditioning (Ipc) was done by three cycles of 2 min ischemia followed by 5 min reperfusion period, and then left renal pedicle was clamped for 45 min. 5) Melatonin-pretreated renal ischemia-reperfusion injury (IRI+M) group: Rats were injected IP with 10 mg/kg BW of melatonin, daily at 8 a.m. for 4 weeks, then the left renal pedicle was clamped for 45 min. 6) Melatonin-pretreated renal ischemia-reperfusion injury with ischemic preconditioning (IRI+Ipc+M) group: Rats were injected with melatonin as in the IRI+M group, and then, they were exposed to Ipre as described in IRI+Ipc group before the definitive 45 min ischemia.

***Blood sampling and assay***

After 12 hr of fasting, blood samples were collected from the retro-orbital venous plexus of rats (13). Each sample was left for clotting for 10 min and centrifuged at 4000 rpm for another 10 min to isolate the serum, which was stored at -20 °C for analysis of serum creatinine and blood urea nitrogen (BUN).

Thereafter, rats were anesthetized with IP injection of a high dose of thiopental sodium (12 mg/100 g BW). A midline laparotomy was made and the left kidney was removed and bisected longitudinally into two halves. One half of the kidney was exposed to tissue homogenization for measurement of the kidney proinflammatory cytokine, tumor necrosis factor-alpha (TNF-α), the anti-inflammatory cytokine, interleukin-13 (IL-13), the apoptotic protein caspase-3, total nitrite/nitrate, and malondialdehyde (MDA) levels, as well as the activities of the antioxidant enzymes, glutathione peroxidase (GPx) and superoxide dismutase (SOD). The other half was placed and fixed in 10% formalin solution and was stained with Haematoxylin & Eosin (H&E) for histopathological examination.

***Biochemical analysis***

Serum creatinine and BUN levels (mg/dl) were determined by the enzymatic colorimetric method using test reagent kits (Bio-diagnostic Company, Egypt) according to the manufacturer’s instructions. Renal tissue homogenate TNF-α level (pg/mg.protein) was determined using an ELISA kit purchased from Abnova Company (R&D systems, USA). IL-13 level (pg/mg.protein) was determined using its specific ELISA kit (Abcam, USA). The apoptotic protein caspase-3 activity in renal tissue (U/g.tissue) was determined by ELISA (Abcam, USA). Homogenate nitrite/nitrate and MDA levels (nmol/g.tissue) were measured by colorimetric methods, using specific kits from Bio Diagnostics Company (Egypt). The activities of GPx and SOD in kidney tissue (U/g.tissue) were determined by colorimetric methods (Bio Diagnostics Company, Egypt).

***Melatonin preparation***

Hundred mg of melatonin powder (Bio Basic, Canada) was dissolved in 1 ml of absolute ethanol and mixed up with one liter of tap water to give a concentration of 0.1 mg/ml. The solution of melatonin was freshly prepared three times a week. The bottles with melatonin solution were covered with a dark foil. The melatonin dosage used provided approximately 10 mg/kg/day (14). 

***Statistical analysis***

The data were presented as the mean ± standard deviation (SD) in each experimental group. Data were analyzed by one-way analysis of variance (ANOVA) and *post hoc* least significant difference was calculated using SPSS (Statistical Package for the Social Science; SPSS Inc., Chicago, IL, USA) version 22. The significant level was set on probability *P*≤ 0.05 (15). 

Histopathological data were analyzed by Chi-square test.

## Results

The sham group showed an insignificant difference (*P*>0.05) in all the measured parameters when compared with that in the Control group in all parameters.

***Renal function tests (creatinine and BUN)***

Creatinine levels (mg/dl) in IRI, IRI+Ipc, IRI+M, and IRI+Ipc+M groups were significantly increased (*P*<0.001) when compared with that of the C group. The creatinine level of the IRI+Ipc+M group was significantly lower (*P*<0.001) than those of the IRI, IRI+Ipc, and IRI+M groups. There was an insignificant difference (*P*=0.53) in creatinine levels between IRI and IRI+Ipc groups (Table 1).

BUN levels (mg/dl) in IRI, IRI+Ipc, IRI+M, and IRI+Ipc+M groups were significantly increased (*P*<0.001) when compared with that of the C group. The BUN level of the IRI+Ipc+M group was significantly lower (*P*<0.001) than those of the IRI, IRI+Ipc, and IRI+M groups (Table 1). 

***Pro-inflammatory and anti-inflammatory cytokines***

TNF-α levels (pg/mg.protein) in IRI, IRI+Ipc, IRI+M, and IRI+Ipc+M groups were significantly increased (*P*<0.001) when compared with that of the C group. TNF-α level of the IRI+Ipc+M group was significantly lower (*P*<0.001) than those of the IRI, IRI+Ipc, and IRI+M groups (Table 2).

IL-13 levels (pg/mg.protein) in IRI, IRI+Ipc, IRI+M, and IRI+Ipc+M groups were significantly decreased (*P*<0.001) when compared with that of the C group. IL-13 level of the IRI+Ipc+M group was insignificant changed (*P*=0.15) when compared with that of the IRI+M group but was significantly higher (*P*<0.001) than those of IRI and IRI+Ipc groups (Table 2).

***The apoptotic protein***

Caspase-3 activities (U/g.tissue) in IRI, IRI+Ipc, IRI+M, and IRI+Ipc+M groups were significantly elevated (*P*<0.001) when compared with that of the C group. Caspase-3 activity of the IRI+Ipc+M group was significantly lower (*P*<0.001) than those of the IRI, IRI+Ipc, and IRI+M groups (Table 2).

***Changes in oxidative stress markers***

Total nitrite/nitrate levels (nmol/g.tissue) in IRI, IRI+Ipc, IRI+M, and IRI+Ipc+M groups were significantly increased (*P*<0.001) when compared with that of the C group. The nitrite/nitrate level of the IRI+Ipc+M group was significantly lower (*P*<0.001) than those of the IRI, IRI+Ipc, and IRI+M groups. There was an insignificant difference (*P*=0.5) in nitrite/nitrate levels between IRI+Ipc and IRI+M groups (Table 3).

MDA levels (nmol/g.tissue) in IRI, IRI+Ipc, IRI+M, and IRI+Ipc+M groups were significantly increased (*P*<0.001) when compared with that of the C group. The MDA level of the IRI+Ipc+M group was significantly lower (*P*<0.001) than those of the IRI, IRI+Ipc, and IRI+M groups. There was insignificant difference (*P*=0.99) in MDA levels between IRI+Ipc and IRI+M groups (Table 3).

GPx activity (U/g.tissue) in IRI and IRI+Ipc groups was significantly decreased (*P*<0.001), when compared with that of the C group. There was insignificant variation (*P*>0.05) between other comparisons (Table 3).

SOD activity (U/g.tissue) in IRI, IRI+Ipc, IRI+M, and IRI+Ipc+M groups was significantly decreased (*P*<0.001) when compared with that of the C group. SOD activity of the IRI+Ipc+M group was insignificantly changed (*P*=0.27), when compared with that of the IRI+M group but was significantly higher (*P*<0.001) than those of the IRI and IRI+Ipc groups. There was an insignificant difference (*P*=0.87) in SOD activity between IRI+Ipc and IRI+M groups (Table 3).

***Histopathological examination***

**Figure 1:**
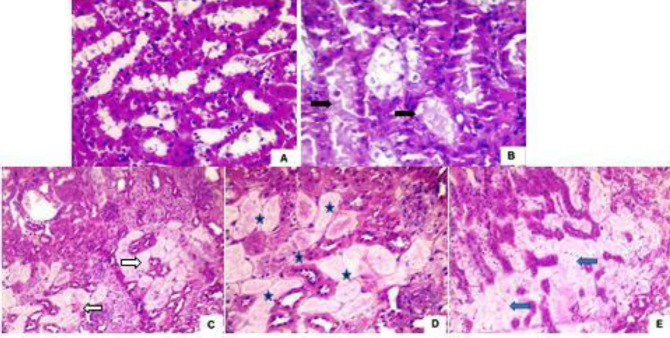
Histopathological assessment of rat left kidney showing the degrees of renal tubular damage (9). (A) showing no change with neither necrosis of tubular epithelium nor cast formation in tubular lumina, score 0; (B) showing individual cell necrosis () with pyknotic nuclei and cytoplasmic vacuolization, score 1; (C) showing complete tubular necrosis with total loss of nuclei and tubular brush border () less than 25%, score 2; (D) showing tubular necrosis () between 25% and 50%, score 3; (E) showing tubular necrosis () more than 50%, score 4 (examined under a light microscope; Olympus bx41, H&E x200 for all)

The six studied groups were performed, according to the grading scale (9); score (0): no change, score (1): unicellular patchy isolated necrosis, score (2): tubular necrosis less than 25%, score (3): tubular necrosis between 25 and 50%, score (4): tubular necrosis more than 50%. In the C group, 100% of rats scored 0. In the S group, 87.5% scored 0 while 12.5% scored 1. 37.5% of rats in the G3 IRI group were scored 3, and 62.5% were scored 4. In the IRI+Ipc group, 37.5% of rats scored 2 and 62.5% were scored 3. Half of the rats in the IRI+M group were of score 3 and the other half were of score 4. In the IRI+Ipc+M group, half of the rats scored 1 while the other half scored 2 (Table 4 and Figure 1).

## Discussion

The reduction in renal function after renal IRI, observed in our study by elevation of serum creatinine and BUN levels and histological damage, are supported with the findings of Bussmann *et al*. (16): serum values of creatinine are directly proportional to the increase in the severity of the renal injury. Also, Souza *et al*. (17) found that IRI significantly increased creatinine and urea concentrations, showing that IRI had deleterious effects on renal function.

It is well known that organ ischemic injury and the resulting necrosis have the potential to activate a prompt immune response, which further induces an inflammatory reaction, and release of free radicals that ultimately lead to subsequent notable organ damage (18).

Renal IRI triggers an inflammatory cascade that is involved in more renal damage, so inhibition of inflammatory responses is a therapeutic approach to protect renal tissue (19). Chemokines are major mediators of the inflammation that regulate pro-inflammatory cytokines, adhesion molecule expression, and leukocyte infiltration and activation (20). Pro-inflammatory cytokines, such as IL6 and TNF-α, play a major role in renal dysfunction of IRI (21). 

The present work shows that renal IRI increased pro-inflammatory cytokines and decreased anti-inflammatory cytokines. The increase of TNF-α after IRI was also reported by Tuğtepe *et al*. (22), who found that increased TNF-α accompanies the elevation of urea and creatinine concentrations. In agreement, Yang *et al*. (23) observed an increase in TNF-α in IRI animals versus sham-operated ones. 

Our data show that the activity of the apoptotic protein caspase-3 in rats exposed to IRI was significantly higher than that in normal rats; a finding that proves the results of another study (24).

In the present study, high kidney tissue total nitrite/nitrate level in IRI indicates increased expression of iNOS. In consistency, Ferdinandy and Schulz (25) stated that IRI could increase significantly the expression of iNOS**.** Other studies have suggested that the increased NO_, _via iNOS activity during renal ischemia, is deleterious to the kidney, and inhibition of iNOS before IRI has dramatic functional protection of kidneys against ischemic renal injury (26). Melatonin prevented iNOS activation and reduced the concentration of NO; this is related to its antioxidant activity. Our results were consistent with another study (27), where they found that melatonin prevented iNOS activation.

Our observation of increased kidney MDA after IRI is in agreement with the previous study of Sener *et al*. (28), in which elevated levels of lipid peroxidation products were increased from 40 to 100% above basal values**.**

The reduction of GPx and SOD activities after renal IRI was also noticed by other researchers (29) who think that the activities of the antioxidant enzymes during IRI are determined by several factors, including the magnitude of IRI, the duration of IR periods, and the specific organ subjected to IRI. 

Our finding was also in harmony with those of two other studies (30, 31) where they found decreased GPx activity in rats subjected to 45 min ischemia and 24 hr reperfusion. In fact, IR causes accumulation of free radicals and reduction of antioxidant enzymes, which have deleterious effects on the cell membrane, DNA, and protein. SOD and catalase activities decrease markedly during renal ischemia, with the main factor being the time of exposure to ischemic insult (32). 

The IRI-impaired renal functions were improved after melatonin pretreatment, as characterized by reduced renal function tests, decreased oxidative stress markers, and less histological damage. Our histopathological findings were in agreement with Souza *et al*. (17), who revealed that pre-treatment with melatonin alone slightly attenuated the histopathological damages after IR, with no kidney experiencing grade 4 injury, but the damage did not differ markedly from that in the control group. Melatonin has been shown to act as a cytoprotective agent in IR-induced lesions, reversing the damage caused by IR nephrotoxicity (17). Also, Sener *et al*. (28) reported that melatonin has protective effects on IR-induced renal injury and the histopathological changes are reversed by melatonin treatment. They (28) proposed that melatonin appears to play a cytoprotective role in the kidney insulted by IR. Supporting this proposal, it was realized that melatonin has protective effects on glomerular and tubular function. Melatonin severely attenuated the histopathological changes; the nearly normal renal tissue structure was preserved by melatonin pretreatment. This cytoprotective effect of melatonin may be due to its powerful antioxidant properties (28).

Studies have shown the beneficial effects of free radical scavengers and antioxidants on IRI (33). Oxygen-free radical-mediated renal damage during the reperfusion period following ischemia was prevented by free radical scavengers and antioxidant activities of melatonin (34). So supplementations with antioxidant agents have protective effects in IRI-induced oxidative stress. 

The present study was in agreement with findings showing that melatonin activates SOD and GPx after IRI (35). The present results were in agreement with Alzahrani (18) who found that melatonin pretreatment improved kidney damage with decreased MDA levels and increased SOD activity.

Research (36) showed that administration of melatonin preserved renal function, and the protective effect was associated with ameliorated oxidative stress, limited pro-inflammatory cytokine production, and neutrophil and macrophage infiltration. Moreover, autophagic flux was increased after melatonin administration, while the apoptosis levels were decreased in the melatonin-pretreated mice. Using TAK-242 and CRX-527, they (36) confirmed that MyD88-dependent TLR4 and MEK/ERK/mTORC1 signaling participated in melatonin-induced autophagy in IR mice. Collectively, the results provide novel evidence that antecedent melatonin treatment provides protection for the kidney against IRI by enhancing autophagy, as regulated by the TLR4/MyD88/MEK/ERK/mTORC1 signaling pathway. Therefore, melatonin preconditioning offers a potential therapeutic approach to prevent renal IRI related to high-risk renal diseases (36).

The kidney can be preconditioned by a non-lethal period of ischemia, which makes it tolerant to subsequent ischemia-induced injury (37). In a study, renal Ipc reduced cell lysis, apoptosis, and lipid peroxidation with the improvement of renal function in the ischemic 

kidney (38). Reduction of adhesion molecules and inflammatory responses may be the mechanism of Ipc-preventing effects (39). However, in other studies, Ipc appears to be mediated via pre-ischemic activation of adenosine receptors, specifically A1 adenosine receptors (40).

A study (41) demonstrated that the protective effects of Ipc include two distinct phases: the early phase, lasting 2 to 3 hr, is not dependent on protein synthesis, but rather requires activation of adenosine receptors and ATP-sensitive potassium channels, while the late phase, beginning 12 to 24 hr after the initial insult, persists for several days and is associated with endothelial progenitor cell mobilization and recruitment. The late phase was extended up to 7 days with significantly lower creatinine and BUN values in Ipc compared with the non-preconditioned ischemic controls (41).

Tossy *et al*. (42) reported that an Ipc regimen, applied 5 min before a sustained ischemic insult to the kidney, confers clear functional, and probably structural, protection to the organ, as determined 2 days after the procedures (42).

In the present study, combined melatonin pretreatment and Ipc have a better beneficial effect than those of using each alone, as proved by more decrease in creatinine and BUN levels, increase in the anti-inflammatory cytokine IL-13 and the anti-oxidant enzyme activities of GPx and SOD, with better improvement of the kidney’s histopathological picture.

**Table 1:** Serum creatinine and blood urea nitrogen (BUN) levels in control group (C), sham operation group (S), and renal ischemia-reperfusion groups with no pretreatment (IRI), ischemic preconditioning (IRI+Ipc), melatonin-pretreatment (IRI+M), or both preconditioning and melatonin pretreatment (IRI+Ipc+M)

Parameters	C	S	IRI	IRI+Ipc	IRI+M	IRI+Ipc+M	ANOVA F test	*P*- value
Creatinine (mg/dl)	0.64 ±0.05	0.7 ±0.08	1.5 ±0.09*†	1.44 ±0.07*†	1.17 ±0.08*†‡§	0.99 ±0.06*†‡§¦	181.34	<0.001
BUN (mg/dl)	15.5 ±0.53	18.91 ±2.19	86.54 ±2.38*†	63.54 ±5.06*†‡	50.9 ±0.73*†‡§	41.97 ±1.67*†‡§¦	883.78	<0.001

Results are represented as mean ± SD. Number of rats in each group was eight (n=8). Statistical analysis was done by one-way ANOVA. The significance of the *P*-value was set at *P*≤0.05. The marks *, †, ‡, §, and ¦ indicate significant differences, when values are compared with the corresponding values of C, S, IRI, IRI+Ipc, and IRI+M groups, respectively

**Table 2:** Kidney homogenate tumor necrosis factor-α (TNF-α), interleukin-13 (IL-13) levels and caspase-3 activity in control group (C), sham operation group (S), and renal ischemia-reperfusion groups with no pretreatment (IRI), ischemic preconditioning (IRI+Ipc), melatonin-pretreatment (IRI+M), or both preconditioning and melatonin pretreatment (IRI+Ipc+M)

Parameters	C	S	IRI	IRI+Ipc	RI+M	IRI+Ipc+M	ANOVA F test	*P*- value
TNF-α (pg/mg.protein)	8.92 ±0.62	9.98 ±0.74	27.16 ±1.18*†	23.39 ±0.78*†‡	17.81 ±1.09*†‡§	15.88 ±1.76*†‡§¦	347.26	<0.001
IL-13 (pg/mg.protein)	49.75 ±5.17	47 ±1.69	22.25 ±2.55*†	30.12 ±1.81*†‡	35 ±2.07*†‡§	38.37 ±1.06*†‡§	106.42	<0.001
Caspase 3 (U/g.tissue)	512.25 ±8.28	542.25 ±15.04	1838 ±33.92*†	1238.13 ±4.49*†‡	1134 ±31.84*†‡§	836.63 ±33.79*†‡§¦	3312.1	<0.001

Results are represented as mean ± SD. Number of rats in each group was eight (n=8). Statistical analysis was done by one-way ANOVA. The significance of the *P*-value was set at *P*≤0.05. The marks *, †, ‡, §, and ¦ indicate significant differences, when values are compared with the corresponding values of C, S, IRI, IRI+Ipc, and IRI+M groups, respectively

**Table 3:** Kidney homogenate total nitrite/nitrate and malondialdehyde levels, and glutathione peroxidase and superoxide dismutase activities in control group (C), sham operation group (S), and renal ischemia-reperfusion groups with no pretreatment (IRI), ischemic preconditioning (IRI+Ipc), melatonin-pretreatment (IRI+M), or both preconditioning and melatonin pretreatment (IRI+Ipc+M)

Parameter	C	S	IRI	IRI+Ipc	IRI+M	IRI+Ipc+M	ANOVA F test	*P*-value
Nitrite/nitrate (nmol/g.tissue)	22 ±1.39	24.5 ±2.14	51.12 ±2.37*†	43.25 ±1.49*†‡	41.69 ±1. 85*†‡	35.38 ±1.06*†‡§¦	350.66	<0.001
MDA (nmol/g.tissue)	6.69 ±1.07	7.86 ±2.74	22.22 ±0.69*†	14.15 ±2.01*†‡	13.7 ±0.91*†‡	10.82 ±1.27*†‡§¦	71.201	<0.001
GPx (IU/g.tissue)	119.15 ±71.9	111.03 ±8.64	47.08 ±4.19*†	68.31 ±4.95*	79.16 ±6.48	83.45 ±7.54	6.41	<0.001
SOD (U/g.tissue)	24.25 ±0.003	23.38 ±0.003	6.88 ±0.001*†	14.25 ±0.001*†‡	15.56 ±0.002*†‡	18.13 ±0.002*†‡§	58.98	<0.001

Results are represented as mean ± SD. Number of rats in each group was eight (n=8). Statistical analysis was done by one-way ANOVA. The *P*-value significant level was set at *P*≤0.05. The marks *, †, ‡, §, and ¦ indicate significant differences, when values are compared with the corresponding values of C, S, IRI, IRI+Ipc, and IRI+M groups, respectively

**Table 4:** Histopathological assessment of control group (C), sham operation group (S), and renal ischemia-reperfusion groups with no pretreatment (IRI), ischemic preconditioning (IRI+Ipc), melatonin-pretreatment (IRI+M), or both preconditioning and melatonin pretreatment (IRI+Ipc+M)

***P*** **-value**	**IRI+Ipc+M**	**IRI+M**	**IRI+Ipc**	**IRI**	**S**	**C**	**Histopathological score**
**<0.001**					7 (87.5%)	8(100%)	Score 0
	4 (50%)				1 (12.5%)		Score 1
	4 (50%)		3 (37.5%)				Score 2
		4 (50%)	5 (62.5%)	3 (37.5%)			Score 3
		4 (50%)		5 (62.5%)			Score 4

Number of rats in each group was eight (n=8). The Chi-square test (x^2) is used to compare between groups regarding the histopathological score

## Conclusion

Isolated melatonin pretreatment or Ipc improves IRI-induced renal injury by protecting kidney tissue against oxidative damage and inflammation. Combined melatonin pretreatment and Ipc has a better beneficial impact than when each is used alone, possibly achieved by an additive effect. Our findings provide novel insights into the potential therapeutic benefits of melatonin in renal IRI. Further studies are needed to investigate how melatonin influence causes IRI, and to clarify the exact mechanisms mediating the effect of Ipc and melatonin combination therapy in renal IRI.

## Authors’ Contributions

HAAR, MSR, GSA, MAK, AMA, and SSD Designed the experiments; AMA Performed experiments and collected data; HAAR, MSR, GSA, MAK, and SSD Discussed the results and strategy; HAAR Supervised, directed and managed the study; HAAR, MSR, GSA, MAK, AMA, and SSD Final approved of the version to be published.
